# Sexualized Drug Use Among Female Sex Workers from Eight Cities in China: A Cross-Sectional Study

**DOI:** 10.1007/s10508-021-02117-2

**Published:** 2021-11-16

**Authors:** Jason J. Ong, Mingzhou Xiong, Joseph D. Tucker, Yajie Wang, M. Kumi Smith, Weiming Tang, Hongyun Fu, Heping Zheng, Bin Yang, Cheng Wang

**Affiliations:** 1 Central Clinical School, Monash University, Melbourne, VIC Australia; 2 Dermatology Hospital of Southern Medical University, No. 2 Lujing Road, Yuexiu District, Guangzhou, 510095 China; 3 Southern Medical University Institute for Global Health and Sexually Transmitted Diseases, Guangzhou, Guangdong China; 4 Faculty of Infectious and Tropical Diseases, London School of Hygiene and Tropical Medicine, London, UK; 5 Division of Epidemiology and Community Health, University of Minnesota, Twin Cities, MN USA; 6 Division of Community Health and Research, Eastern Virginia Medical School, Norfolk, VA USA

**Keywords:** HIV, Female sex workers, Sexualized drug use

## Abstract

There is a rich literature on sexualized drug use (i.e., drug use before or during sex) for men who have sex with men but less data from female sex workers (FSW), particularly from low- and middle-income countries. We describe the sexual and reproductive health outcomes in FSW reporting sexualized drug use. In 2019, we conducted a cross-sectional study in eight cities from seven provinces in China. We recruited FSW through community organizations working with sex workers and included those aged 18 years or above, exchanged sex at least once for money or goods in the past three months, and had traded sex for longer than a year. Multivariable logistic regression models were used. In total, 650 women participated: average age was 38.8 years (SD 10.2), 57.1% reported a monthly income over 5000 RMB ($USD 707), and 12.8% completed high school or above. Among participants, 65 (10.0%, 95% confidence interval (CI) 7.8–12.6) reported a history of sexualized drug use. Compared to FSW who never reported a history of sexualized drug use, FSW who reported a history of sexualized drug use had greater odds of working for a manager compared to being self-employed (adjusted odds ratio (AOR) 4.04, 95% CI 2.12–7.69), work in a sauna (AOR 2.43, 95% CI 1.09–5.41), charging a higher price for vaginal sex (AOR 2.15, 95% CI 1.14–4.06), and ever diagnosed with STIs (AOR 4.51, 95% CI 2.61–7.80). One in ten FSW reported sexualized drug use. Although they had similar risk profiles in terms of consistency of condom use and reproductive health outcomes, these women were more likely to report past STIs than those who reported no sexualized drug use. Health workers who work with substance users should devote attention to the sexual practices of their clients to make sure that they have safer sex.

## Introduction

Female sex workers (FSW) are vulnerable to social and cultural marginalization, stigma and discrimination, socioeconomic disadvantage, and human right abuses (UNAIDS, 2014). This has been further exacerbated during the COVID-19 pandemic (UNAIDS, 2020). FSW are often at higher risk of acquiring HIV/sexually transmitted infections (STIs) than the rest of the population (Baral et al., 2012; Zhang et al., 2020). This elevated risk for acquiring HIV/STIs is exacerbated by the use of illicit drugs, particularly when multiple layers of stigma exist related to sex work, illicit drugs, and STIs (Whitaker et al., 2011). However, public health authorities often overlook this challenging aspect of disease prevention in FSW—illicit drug use. Drug dependence may lead to entry into sex work, used to cope with challenges associated with sex work or as a means to purchase drugs (Gossop et al., 1995; Syvertsen et al., 2019; Ulibarri et al., 2013). Illicit drug use among FSW is associated with mental health issues (such as depression), increased frequency of condomless sex, acquisition of HIV/STIs, unintended pregnancy, violence, and coercive sex (Chen et al., 2013; Church et al., 2001; Luchters et al., 2016; Pandiyan et al., 2012; Semple et al., 2016). Additionally, injecting drug use may increase the risk of acquiring HIV and other blood-borne infections (such as hepatitis C) among FSW (Nguyen et al., 2004; Tran et al., 2005).

Although there is some research about recreational drug use before or during sex—referred to here as *sexualized* drug use—in FSW living in low- and middle-income countries (Li et al., 2014; Loza et al., 2010; Morris et al., 2011; Parry et al., 2009; Shannon et al., 2008; Strathdee et al., 2008; Syvertsen et al., 2019), it is not clear whether FSW who report sexualized drug use may also be at elevated risk for acquiring HIV/STIs; this is in contrast to the rich literature on sexualized drug use for men who have sex with men (Bourne et al., 2018). Regarding sexualized drug use among FSW, studies from the US-Mexican border report sexualized drug use among FSW ranged from 14 to 63% (Loza et al., 2010; Morris et al., 2011; Strathdee et al., 2008). A study of 198 survival sex workers in Canada reported that 59% shared drugs with clients in the preceding six months and that sexualized drug use was associated with inconsistent condom use, verbal harassment, physical and/or sexual assault (Shannon et al., 2008). A qualitative study of Kenyan FSW reported how drugs enabled these women to undertake sex work—‘sex work was intolerable when sober’ (Syvertsen et al., 2019). A study from China reported reasons for sexualized drug use related to client request or the opportunity to make more money (Li et al., 2014). Another study from drug using commercial sex workers (71% females) living in South Africa reported that drugs were used to enhance the sexual experience and prolong sex and may be associated with inconsistent condom use (Parry et al., 2009). Together, these studies highlight the need to understand better how sexualized drug use impacts the health of FSW, especially in contexts (like China) where punitive environments exist for both sex work and drug use, meaning that FSW who engage in sexualized drug use might be at an even greater disadvantage. Drug users and sex workers in China can be detained for up to 15 days. According to the Anti-Drug Law of the People’s Republic of China, drug users can be fined up to 2000 RMB (about 300 US dollars), and individuals may be sent to a drug rehabilitation center for up to 2 years. According to the Law of the People’s Republic of China on Public Security Administration Penalties, sex workers can be fined up to 5000 RMB (about 750 US dollars) depending on the severity of the case.

The Syndemic Model of Health suggests that there may be a clustering of harmful health behaviors due to synergistic interactions between diseases and their social context (Singer et al., 2017). Therefore, FSW reporting sexualized drug use may be more likely to report other harmful health outcomes. Our study aims to contribute to the data of sexualized drug use among FSW in low- and middle-income countries by describing the sociodemographic characteristics, sexual behaviors, and HIV/STI testing behaviors among FSW reporting sexualized drug use. We hypothesized that disaggregating data by sexualized drug use provides valuable insights into their unique health challenges. Specifically, FSW who reported a history of sexualized drug use would have a different risk profile than FSW who reported no sexualized drug use.

## Method

### Participants

This cross-sectional study, led by a provincial STI Control Center, was implemented in eight cities (Beijing, Tianjin, Shenzhen, Kunming, Jiaozhou, Yunfu, Xiangyang and Longnan) within seven provinces in China between August 17 and October 17, 2019. We collaborated with eight community-based organizations (CBOs) in these cities with experience in conducting female sex workers outreach programs (Appendix 1). Eligible participants were aged 18 years or above; exchanged sex (vaginal, oral, and/or anal) at least once for money or goods in the past three months; and willing to participate in the survey by providing verbal inform consent. We restricted the study’s analysis to participants who traded sex for more than a year to capture data from more established workers.

### Procedure

The Wenjuanxing platform (Changsha Haoxing Information Technology Co., Ltd., China) was used to create an online survey link that allowed participants to complete the survey by smartphone or computer tablets. The survey was created by the research group and revised by local CBO stakeholders, policymakers, and international HIV/STI experts. Following a pilot study among 50 FSWs, the survey procedures were adjusted to simplify administration and improve survey comprehensibility. The primary outcome of this study was sexualized drug use among FSW.

The outreach workers of the CBOs recruited FSW using convenience sampling from a variety of workplaces of FSWs: hair salons, saunas, bath centers, karaoke bars, dancing hall bars, sidewalk snack vendors, hotels, nightclubs, massage parlors, guesthouses, and public outdoor places. Interested participants clicked on a survey link that assessed their eligibility, and the research system (Wenjuanxing) verified the uniqueness of the mobile phone number to avoid participants giving multiple entries. Phone numbers of participants were not available to the researchers. Each survey was self-administered by the eligible participant on their device at the recruitment site; they could ask the CBO worker for help if needed.

### Measures

We asked about sexualized drug use through the question: “Have you ever used the following substances before or during sex” (none, heroin, cocaine, crack, methamphetamine, speedball (heroin plus cocaine), other opioids, solvents/glue, hallucinogens, marijuana, other). The survey also asked about: (1) sociodemographic characteristics (age, marital status, monthly income, education level, source of sample, household registration, and migration status); (2) Sexual behaviors (employment status for commercial sex, duration of commercial sex, number of clients served in the past month, average amount received for vaginal sex with a client, consistency of condom use with vaginal sex in the past month, condom use at last vaginal sex, consistency of condom use with oral sex in the past month, consistency of condom use with anal sex in the past month, ever had an unintended pregnancy, ever had an abortion and current contraception method); and (3) HIV/STI testing behaviors (ever had an HIV test, result of last HIV test, ever had syphilis test, result of last syphilis test, ever diagnosed with STIs).

### Statistical Analysis

Data were downloaded from the Wenjuanxing platform and analyzed using IBM SPSS^®^ version 24.0. Descriptive statistics were used to summarize the characteristics of the study sample. *χ*^2^ test or Fisher’s exact test (for categorical variables), and *t* test or Wilcoxon rank-sum test (for continuous variables) were used to identify statistically significant differences in sexual and reproductive health behaviors and outcomes among different groups of FSWs by background characteristics. Multivariable logistic regression models were used to explore the associations between sexualized drug use and sexual behavioral and HIV/STI testing behavioral factors. Based on our literature review, we adjusted for the potential confounders of injecting drug use in the preceding six months (a known risk factor for adverse health outcomes for FSW), age, marital status, migration status, income, and education in each of the models.

## Results

### Sociodemographic Characteristics (Table 1)

**Table 1:** Comparison of the sociodemographic characteristics of female sex workers in China, 2019 (*N* = 650)

Characteristics	Sexualized drug use *n* (%)	No sexualized drug use *n* (%)	*p* value	Total *n* (%)
Total	65 (10.0)	585 (90.0)		
*Age (years)*				
Mean ± SD	33.9 ± 8.2	39.3 ± 10.3	< .001	38.8 ± 10.2
*Marriage*			.001	
Never married and not cohabiting	7 (10.8)	57 (9.7)		64 (9.9)
Never married but cohabiting	9 (13.9)	59 (10.1)		68 (10.5)
Married	41 (63.1)	251 (42.9)		292 (44.9)
Divorced or widowed	8 (12.3)	218 (37.3)		226 (34.8)
*Monthly income* ^a^			.07	
less than 3000 RMB ($USD 424)	6 (9.2)	76 (13.0)		82 (12.6)
3001–5000 RMB ($USD 425–707)	27 (41.5)	170 (29.1)		197 (30.3)
5001-8000 RMB ($USD 708–1131)	18 (27.7)	136 (23.3)		154 (23.7)
Over 8000 RMB (over $USD 1131)	14 (21.5)	203 (34.7)		217 (33.4)
*Education*			.03	
Elementary school or below	27 (41.5)	260 (44.4)		287 (44.1)
Junior high school	23 (35.4)	257 (43.9)		280 (43.1)
High school or above	15 (23.1)	68 (11.6)		83 (12.8)
*Source of sample*			.001	
Foot-washing room/hair salon	18 (27.7)	244 (41.7)		262 (40.3)
Sauna/bath center	17 (26.2)	72 (12.3)		89 (13.7)
Karaoke/dancing Hall/bar	16 (24.6)	87 (14.9)		103 (15.9)
Street	3 (4.6)	95 (16.2)		98 (15.1)
Others^b^	11 (16.9)	87 (14.9)		98 (15.1)
*Household registration*			.89	
Rural	48 (73.9)	434 (74.2)		482 (74.2)
Urban	17 (26.2)	149 (25.5)		166 (25.5)
I am not sure	0 (0)	2 (0.3)		2 (0.3)
*Migration status*			< .001	
Local residents	35 (53.9)	112 (19.2)		147 (22.6)
Another city of this province	18 (27.7)	146 (25.0)		164 (25.2)
Another province	12 (18.5)	327 (55.9)		339 (52.2)

RMB = renminbi or Chinese yuan, 1 RMB = 0.14 US dollar; SD = standard deviation; USD = US dollar

^a^This relates to the overall personal monthly income of the individual and is not restricted to income from commercial sex work. An average worker in China earns approximately $376 USD per month (Chinese National Bureau of Statistics: National Bureau of Statistics. Personal income and consumer spending in 2019 2020. http://www.stats.gov.cn/tjsj/zxfb/202001/t20200117_1723396.html. Accessed 22-April-2021)

^b^Others include sidewalk snack vendor/diner, hotel, nightclub, massage parlor, call girl, online advertising

Overall, their average age was 38.8 years (standard deviation (SD) 10.2), a significant proportion was married (44.9%), about half reported a monthly income over 5000 RMB (US$ 707, 57.1%) and a minority completed high school or above (17.4%, Table 1). FSWs were recruited from a variety of venues: foot-washing rooms/hair salons (40.3%), saunas (13.7%), karaoke/dancing hall/bars (15.9%), street (15.1%), and other venues (15.1%). Most reported a rural household registration (74.2%, an official record of the person’s residential origin), and only a minority were current residents (22.6%).

There were significant differences for sociodemographic variables comparing those who reported a history of sexualized drug use with those who reported no sexualized drug use. Compared to FSW who reported no sexualized drug use, those who reported a history of sexualized drug use were more likely to be younger, married, less monthly income, higher education level, work in a sauna or karaoke bar, have a rural household registration and local residents.

Among 650 participants, 65 (10.0%, 95% CI 7.8–12.6) reported a history of sexualized drug use. Methamphetamine was most commonly used before or during sex (*n* = 24), followed by marijuana (*n* = 16), hallucinogens (*n* = 12), heroin (*n* = 10), and cocaine (*n* = 8). Another 21 participants also reported other drug use beyond our list. Among these 21 participants, six used Rush, four used cough syrup, three used ecstasy, two used ketamine, and one used flunitrazepam, three did not know what drug they took, and two participants did not specify the “other” drug.

### Sex Work Parameters (Table 2)

The majority of FSWs were self-employed (58.0%). A minority of women consistently used condoms with clients in the past month for vaginal sex (42.5%), oral sex (15.5%), and anal sex (3.6%). Most reported an unintended pregnancy (64.6%) or had at least one abortion (66.3%), and only one out of four women were using a long-acting reversible contraceptive (27.6%).

**Table 2:** Comparison of the sexual behavioral characteristics of female sex workers in China, 2019 (*N* = 650)

Characteristics	Sexualized drug use *n* (%)	No sexualized drug use *n* (%)	*p* value^a^	Total *n* (%)
*Total*	65 (10.0)	585 (90.0)		
*Employment status*			< .001	
Self-employed/freelance FSW	16 (24.6)	361 (61.7)		377 (58.0)
Work for a manager	48 (73.9)	216 (36.9)		264 (40.6)
Other	1 (1.5)	8 (1.4)		9 (1.4)
*Clients served in the past month*			.05	
< 22 (cutoff is the median)	37 (56.9)	257 (43.9)		294 (45.2)
> 22	28 (43.1)	328 (56.1)		356 (54.8)
*Average amount received per vaginal sex act (USD)*			< .001	
≤ 21 (cutoff is median)	23 (35.4)	353 (60.3)		376 (57.9)
> 21	42 (64.6)	232 (39.7)		274 (42.2)
*Consistency of condom use with vaginal sex in the past month*^a^			.49	
Not always used	40 (61.5)	334 (57.1)		374 (57.5)
Always used	25 (38.5)	251 (42.9)		276 (42.5)
*Consistency of condom use with oral sex in the past month*^a^			.10	
Not always used	33 (94.3)	306 (83.6)		339 (84.5)
Always used	2 (5.7)	60 (16.4)		62 (15.5)
*Consistency of condom use with anal sex in the past month*^a^			.07	
Not always used	5 (83.3)	49 (98.0)		54 (96.4)
Always used	1 (16.7)	1 (2.0)		2 (3.6)
*Ever had an unintended pregnancy*			< .001	
None	18 (27.7)	212 (36.2)		230 (35.4)
1 time	20 (30.8)	169 (28.9)		189 (29.1)
2–3 times	12 (18.5)	173 (29.6)		185 (28.5)
4–5 times	11 (16.9)	25 (4.3)		36 (5.5)
Over 5 times	4 (6.2)	6 (1.0)		10 (1.5)
*Ever had abortion*			< .001	
None	16 (24.6)	203 (34.7)		219 (33.7)
1 time	17 (26.2)	190 (32.5)		207 (31.9)
2–3 times	17 (26.2)	170 (29.1)		187 (28.8)
4–5 times	13 (20.0)	16 (2.7)		29 (4.5)
Over 5 times	2 (3.1)	6 (1.0)		8 (1.2)
*Current contraception method*				
Male condom	46 (73.0)	425 (75.0)	.74	471 (74.8)
Female condom	18 (27.7)	97 (16.6)	.03	115 (18.3)
Oral contraceptive	19 (30.2)	142 (25.0)	.38	161 (25.6)
IUD/injectables/patches	7 (11.1)	167 (29.5)	< .01	174 (27.6)
Spermicide	0 (0)	45 (7.9)	.01	45 (7.1)
Traditional methods (e.g., safety period calculation, herbal tea)	3 (4.8)	21 (3.7)	.68	24 (3.8)

IUD = intrauterine device; USD = US dollar

^a^Denominators are those who reported vaginal sex, oral sex, and anal sex in the last month

Compared to FSW who reported no sexualized drug use, FSW who reported a history of sexualized drug use were more likely to work for a manager, served fewer clients, received more money for vaginal sex, more likely to have an unintended pregnancy and abortion, and less likely to use long-acting reversible contraception.

### HIV/STI Testing Behaviors (Table 3)

The majority of FSW reported ever testing for HIV (93.4%), and 1.9% reported living with HIV. A lower majority reported ever testing for syphilis (84.8%), and 13.7% reported a positive syphilis result with their last test. A minority of FSW (23.9%) reported ever being diagnosed with an STI (including herpes, chlamydia, gonorrhea, warts, and hepatitis). Compared to FSW who reported no sexualized drug use, FSW who reported a history of sexualized drug use were less likely to be living with HIV but more likely to be diagnosed with an STI in the past.

**Table 3:** Comparison of the HIV/STI testing behaviors of female sex workers in China, 2019 (*N* = 650)

Characteristics	Sexualized drug use *n* (%)	No sexualized drug use *n* (%)	*p* value	Total *n* (%)
*Total*	65 (10.0)	585 (90.0)		
*Ever had an HIV test*			.16	
Yes	58 (89.2)	549 (93.9)		607 (93.4)
No	7 (10.8)	36 (6.2)		43 (6.6)
*Result of last HIV test*^a^			.03	
Positive	0 (0.0)	12 (2.1)		12 (1.9)
Negative	56 (86.2)	535 (91.5)		591 (90.9)
Indeterminate	1 (1.5)	0 (0)		1 (0.2)
I don’t know	1 (1.5)	2 (0.3)		3 (0.5)
*Ever had syphilis test*			.69	
Yes	54 (83.1)	497 (85.0)		551 (84.8)
No	11 (16.9)	88 (15.0)		99 (15.2)
*Result of last syphilis test*			.41	
Positive	5 (7.7)	84 (14.4)		89 (13.7)
Negative	48 (73.9)	405 (69.2)		453 (69.7)
Indeterminate	0 (0)	4 (0.7)		4 (0.6)
I don’t know	1 (1.5)	4 (0.7)		5 (0.8)
*Ever diagnosed with STIs*^b^			< .001	
Yes	35 (53.9)	120 (20.5)		155 (23.9)
No	30 (46.2)	465 (79.5)		495 (76.2)

^a^Denominator here is different due to missing data

^b^Includes herpes, chlamydia, gonorrhea, warts, hepatitis

STI = sexually transmitted infection

Table 4 summarizes the regression analyses of FSW. Compared to FSW who never reported any sexualized drug use, FSW who reported a history of sexualized drug use had greater odds of working for a manager, working in a sauna, received more money for vaginal sex and ever diagnosed with STIs in the past.

**Table 4:** Factors related to sexualized drug use among female sex workers in China, 2019 (*N* = 650)

Variables	Sexualized drug use Crude OR (95% CI)	*p* value	Sexualized drug use Adjusted OR^a^ (95% CI)	*p* value
*Employment status for commercial sex work*				
Self-employed	Ref		Ref	
For a manager	**5.01 (2.78–9.05)**	**< .001**	**4.04 (2.12–7.69)**	**< .001**
*Source of work*				
Foot-washing room/hair salon	Ref		Ref	
Sauna	**3.20 (1.57–6.53)**	**.001**	**2.43 (1.09–5.41)**	**.030**
Karaoke/bar	**2.49 (1.22–5.10)**	**.012**	1.79 (0.82–3.90)	.141
Street	0.42 (0.12–1.49)	.182	0.41 (0.12–1.43)	.162
Other	1.71 (0.78–3.77)	.181	1.55 (0.69–3.50)	.289
*Average price for vaginal sex (USD)*				
≤ 21	Ref		Ref	
> 21	**2.78 (1.63–4.74)**	**< .001**	**2.15 (1.14–4.06)**	**.018**
*Current long-acting reversible contraception*				
No	Ref		Ref	
Yes	**0.30 (0.14–0.68)**	**< .001**	0.49 (0.21–1.15)	.100
*Ever diagnosed with STIs*				
No	Ref			
Yes	**4.52 (2.67–7.66)**	**< .001**	**4.51 (2.61–7.80)**	**< .001**

OR = odds ratio; Ref = Reference level; USD = United States Dollar; 95% CI = 95% confidence interval

^a^Adjusted for each variable in the table, as well as injecting drug use, age, marital status, migration status, income, and education

## Discussion

This study of 650 FSW from eight Chinese cities reveals that a history of sexualized drug use identified FSW who had greater associations with adverse sexual and reproductive health outcomes, even after accounting for injecting drug use (a known risk factor for adverse health outcomes in FSW). While much has been published about the associations between injecting drug use and adverse sexual and reproductive health outcomes (Cepeda et al., 2015; Hail-Jares et al., 2016; Lau et al., 2012; Lau et al., 2007; Le et al., 2016; Li et al., 2014; Liao et al., 2016; McDougal et al., 2013; Medhi et al., 2012; Odinokova et al., 2014; Yao et al., 2012; Zhang et al., 2014), our study provides data on sexualized drug use (independent of injecting drug use) among FSW.

The self-report of sexualized drug use among FSW compounded their risks for sexual and reproductive health outcomes compared to their non-drug using peers. It is already established that injecting drug use among FSW is associated with increased risks of STIs (Cepeda et al., 2015; Hail-Jares et al., 2016; Li et al., 2014; Liao et al., 2016; Medhi et al., 2012; Yao et al., 2012), HIV (Le et al., 2016; Li et al., 2014; Yao et al., 2012), higher unintended pregnancy rates (Li et al., 2014), reproductive morbidity (McDougal et al., 2013), less consistent condom use (Lau et al., 2007, 2012; Li et al., 2014), violence (McDougal et al., 2013; Odinokova et al., 2014; Zhang et al., 2014), and mental health problems (Zhang et al., 2014). We add to this literature by demonstrating a history of sexualized drug use is also associated with adverse sexual and reproductive health outcomes. Our disaggregated data of FSW reporting sexualized drug use uncovered significant differences in sociodemographic characteristics, sexual behaviors, and HIV/STI testing behaviors compared with their non-drug using peers. This emphasizes the need to measure sexualized drug use among FSW as an additional risk factor for adverse health outcomes and thus direct additional resources to them such as empowerment-based harm reduction programs (Hawk et al., 2017) or strength-based interventions (Surratt et al., 2014). In China, methadone maintenance clinics, needle exchange programs, drug use counseling, and voluntary drug rehabilitation centers are available in major cities.

We also found that compared to FSW who never reported any sexualized drug use, FSW who reported a history of sexualized drug use had greater odds of working for a manager, working in a sauna, and received more money for vaginal sex. There are complex reasons for sexualized drug use, which could pull or push FSW into using drugs. For instance, a study from Chinese FSW found that client request and monetary incentives were reasons for drug use with clients (Li et al., 2014). This is similar to experiences of FSW from other countries where FSW earned money to sustain their drug habit (Syvertsen et al., 2014) or directly exchange sex for crack (cocaine) (Duff et al., 2013). It is possible that working for a manager or working in a sauna may facilitate the use of sexualized drug use but as this was a cross-sectional study, we were unable to determine the direction of causality. Further qualitative research will be needed to understand this association with the greater likelihood of sexualized drug use.

The main strength of our study is acquiring data from a large number of FSW working in diverse locations, with varying durations of time working in the sex industry. Our data, disaggregated by sexualized drug use, are a unique contribution to the current literature on FSW and highlight the unique challenges faced by a distinct group of sexualized drug-using FSW. Our findings should be read in light of several limitations. Although we attempted to recruit a representative sample of FSW working in diverse settings, this was not a random sample. There is a risk of social desirability bias as we relied on self-report of drug use, consistent with other studies regarding drug use. We tried to mitigate this bias by the anonymous nature of the survey. Our survey question related to drug use during or before sex was not specific for sexualized drug use during commercial sex, nor was it able to determine the pattern of use (e.g., only at the entry into sex work or throughout). Therefore, our findings of the association with adverse health outcomes related to women reporting ever experiencing sexualized drug use (regardless of whether this was in the context of commercial sex work or not, and the pattern of sexualized drug use). We did not include questions regarding alcohol use before or during sex, which may also increase the risk of HIV/STIs in FSW (Wang et al., 2010). Given the brief nature of the survey, we did not ask about how drug use affected women’s lives, and we could not distinguish between non-problematic and problematic drug use. More research is needed to explore the drug-related harms of sexualized drug use for FSW. We did not differentiate between the experiences of FSW who received money from those who received goods for delivering sexual health services. Finally, we did not include males or transgender sex workers who may have an even higher risk for HIV/STIs. This will be the subject of future research.

### Conclusion

Our study highlights the sexual and reproductive health needs of FSW with a history of sexualized drug use. Similar to guidelines regarding sexualized drug use among MSM (Clutterbuck et al., 2018), we recommend additional supports for FSW who report sexualized drug use to mitigate the associations with other adverse sexual and reproductive health outcomes. Additional supports may include greater access to drug counseling, methadone maintenance programs, needle exchange programs, drug rehabilitation services, and compassionate care by health care providers. Similarly, health workers who work with substance users should devote some attention to the sexual practices of their clients to make sure that they have safer sex.

## Data Availability

All data generated or analyzed during this study are included in this published article. Additional information may be requested from the corresponding author.

## Appendix 1

**Table:**

Name of institution	Number of FSW served in 2018	Official cooperation with Government/CDC/Hospital	Primary services provided to FSW	City	Region of China	Population of the city	GPD per capita (USD)	Type of city	Ethnic breakdown
Beijing Firefly Women Group	700	Yes	1. HIV/STD test and treatment promotion 2. High-risk sex behavior intervention	Beijing	Northern	21.54 million	24k	1st tier	Han (95.69%), Manchu, Hui, Mongolian, Korean, etc. 56 ethnics
Tianjin Sunflower Migrant Women Service Center	3000	Yes	1. Training skills on reproductive health and HIV/STD prevention 2. Provide HIV/STD testing 3. Provide HIV/syphilis self-test kit	Tianjin	Northern	15.62 million	17 k	1st tier	Han (97%), Hui (2%), Manchu (0.6%), Mongolian, Korean, etc. 52 ethnics
Xiangyang Ziwei Garden Health Consulting Studio	1200	Yes	1. Training skills on reproductive health and HIV/STD prevention 2. Provide HIV/STD testing 3. Provide HIV/syphilis self-test kit 4. Community empowerment	Xiangyang	Central South	6.05 million	11 k	3rd tier	Han (99.6%), Hui (0.4%)
Shenzhen Hongci Women Care Service Center	5000	Yes	1. Training skills on reproductive health and HIV/STD prevention 2. Provide HIV/STD testing 3. STD linkage to care	Shenzhen	Southern	13.44 million	29.5 k	1st tier	Han, Zhuang, Tujia, Miao, Dong, etc. 56 ethnics (7.8%)
Yunfu Zhongyue AIDS Care Rescue Center	600	Yes	1. High-risk sex behavior intervention 2. Provide psychological support 3. HIV/syphilis/NG consulting and test	Yunfu	Southern	2.55 million	4.7 k	4th tier	99.6% Han, with 0.4% minority ethnicity
Kunming Parallel Sexual Health Support and Development Center	1200	Yes	High-risk sex behavior intervention 2. Provide HIV/STD testing	Kunming	Southwest	6.95 million	10.6 k	2nd tier	Han (86.16%), minority ethnics (13.84%)
Longnan Longjiang Support Group	1940	Yes	1. Provide a platform for FSW to get HIV/syphilis/HCV test	Longnan	Northwest	2.64 million	2.2 k	5th tier	Han (97.69%), minority ethnics (2.31%)
Jiaozhou Love Health Service Center	2000	Yes	1. High-risk sex behavior intervention 2. Gynecological examination 3. Provide HIV/STD testing 4. Training skills on reproductive health and HIV/STD prevention	Jiaozhou	Eastern	0.91 million	18 k	4th tier	Han (99.3%), minority ethnics (0.7%)
